# A Metal Importer and Exporter Interact Differently in the Chloroplast and Cell Membrane

**DOI:** 10.3390/membranes16050167

**Published:** 2026-05-02

**Authors:** Karnelia Paul, Biswajit Ray, Chinmay Saha, Anupam Roy, Sohini Basu, Anindita Seal

**Affiliations:** 1 Department of Biotechnology and Dr. B. C. Guha Centre for Genetic Engineering and Biotechnology, University of Calcutta, 35 Ballygunge Circular Road, Kolkata 700019, India; k1paul@unc.edu (K.P.); brbcg_rs@caluniv.ac.in (B.R.); sahac@unc.edu (C.S.); arbcg_rs@caluniv.ac.in (A.R.); 2 Department of Biochemistry, University of Calcutta, 35 Ballygunge Circular Road, Kolkata 700019, India; basusohini90@gmail.com

**Keywords:** *Brassica juncea*, metal homeostasis, yellow stripe-like, natural resistance-associated macrophage protein

## Abstract

Metal homeostasis, which coordinates the influx and efflux of essential elements such as iron (Fe) and manganese (Mn) in chloroplasts, is essential for optimum photosynthesis, especially in metal-accumulating plants. *Brassica juncea* (Indian mustard) is a metal-tolerant species with a strong metal accumulation capacity, making it a suitable model for studying transition metal homeostasis. In this study, we identified two efflux transporters, BjYSL6.1 and BjYSL6.4, that localize in the endomembrane system of *Schizosaccharomyces pombe* and interact with the chloroplast Mn influx transporter BjNRAMP4.1 at the plasma membrane and within the chloroplasts. Bimolecular fluorescence complementation and split-ubiquitin yeast two-hybrid assays confirmed specific protein–protein interactions among these transporters, as well as with the membrane-bound thioredoxin BjHCF164, a known regulator of photosynthetic electron transport. Gene expression studies revealed that *BjNRAMP4.1* and *BjYSL6* isoforms are inversely regulated under Fe and Mn stress conditions, with *BjNRAMP4.1* being strongly induced under deficiency, whereas *BjYSL6.1* and *BjYSL6.4* are downregulated. These findings suggest that a coordinated network involving BjNRAMP4.1, BjYSL6s, and BjHCF164 modulates metal influx and efflux at the chloroplast and plasma membrane interfaces, thereby maintaining metal homeostasis, which is critical for photosynthetic efficiency in *B. juncea*.

## 1. Introduction

Yellow stripe-like (YSL) transporters belong to a distinct clade within the oligopeptide transporter (OPT) superfamily. The membrane-bound Fe(III)-phytosiderophore (Fe(III)-PS) transporter of maize, Yellow Stripe 1 (*ZmYS1*), was the first functionally characterized member of this family [1,2]. The phytosiderophores (PS) are mugineic acid–family compounds secreted by grasses to chelate Fe(III) in the rhizosphere, whereas nicotianamine (NA) is an internal metal chelator that binds Fe(II) and other divalent metals for intracellular and long-distance transport. *ys1* mutant deficient in the maize YS1 protein exhibits interveinal chlorosis or “yellow stripes” due to impaired utilization of Fe(III)-PS complexes [3,4,5,6]. The *ZmYS1* gene complements the *fet3fet4* yeast mutant, which is defective in Fe uptake, but only in the presence of Fe(III)-PS [1,7], and electrophysiological studies have confirmed Fe(III)-PS transport activity in Xenopus oocytes [8,9].

Although non-grass species do not synthesize or secrete phytosiderophores, multiple YSL genes have been identified in monocots, dicots, gymnosperms, ferns, and mosses, indicating that these proteins have evolved alternative functions. In these plants, YSLs primarily function as Fe(II)-nicotianamine (Fe(II)-NA) transporters, facilitating long-distance iron translocation and distribution within the plant, whereas in grasses, YSLs act as (Fe(III)-PS) transporters mediating iron uptake from the rhizosphere [1,10]. In addition to Fe, YSL proteins have been implicated in the transport of other metals, such as Zn, Cu, and Mn [11,12]. Recent genome-wide studies in *Zea mays* and *Nicotiana tabacum* have expanded the known YSL family and revealed diverse expression patterns across tissues and subcellular compartments, reinforcing the multifaceted roles of YSLs in metal uptake, redistribution, and detoxification [10,13].

Metals are continuously trafficked between extracellular spaces and subcellular organelles to maintain metabolic equilibrium [14]. Importantly, while most YSLs have been characterized as metal–nicotianamine importers, YSL4 and YSL6 represent a distinct functional subgroup that operates as metal efflux transporters. *Arabidopsis* YSL4 and YSL6 localize to the tonoplast and chloroplast envelope, respectively, where they mediate the export of metal-NA complexes from vacuoles or chloroplasts into the cytosol [15,16]. Mutations in *Arabidopsis* YSL4 and YSL6 result in chloroplastic Fe accumulation [16], suggesting that these transporters function as Fe exporters, protecting chloroplasts from metal-induced oxidative damage during germination.

Natural resistance-associated macrophage proteins (NRAMPs) are proton-coupled metal ion transporters that are conserved across prokaryotes and eukaryotes. The first characterized member, NRAMP1, is expressed in mammalian phagosomes during pathogen invasion [17]. In plants, NRAMPs mediate Fe and Mn transport [18,19,20,21] and facilitate the movement of other essential and toxic metals, including Zn, Cu, Co, Ni, Cd, Al, and As [22,23,24,25,26,27,28].

In *Brassica juncea*, a well-known heavy-metal accumulator, BjNRAMP4.1, an ortholog of AtNRAMP4, was previously shown to localize not only to the tonoplast, plasma membrane but also to chloroplasts, where it interacts with the thylakoid membrane-bound thioredoxin-like protein BjHCF164 [29]. The *hcf164* mutant survives only in the heterozygous form and exhibits compromised photosynthesis due to defective redox regulation and cytochrome b6f assembly [30,31,32]. HCF164 also regulates the kinase STN7/STT7, which modulates short- and long-term photosynthetic acclimation [33,34]. The ability of BjNRAMP4.1 and BjHCF164 to interact in both leaf chloroplasts and root membranes suggests that certain transporters may physically associate with redox regulators, potentially linking chloroplast redox balance with metal homeostasis [29].

NRAMP3 and NRAMP4 are critical for mobilizing vacuolar Mn and Fe stores to support chloroplastic photosynthetic demand [20], whereas PAM71 and CMT1 mediate Mn import into the thylakoid lumen and chloroplast stroma, respectively [35,36,37]. The PIC1 permease functions in Fe import across the chloroplast envelope [38].

In this study, we describe the interaction of *B. juncea* metal transporters BjYSL6.1 and BjYSL6.4 with BjNRAMP4.1 and thioredoxin BjHCF164. These interactions occur at both the plasma membrane and chloroplast, suggesting the possibility of coordinated regulation of metal exchange between these compartments during homeostasis. Furthermore, the expression patterns of *BjYSL6.1*, *BjYSL6.4*, and *BjNRAMP4.1* were inversely correlated under Fe and Mn stress, indicating that the opposing regulation of efflux and influx transporters may contribute to maintaining chloroplastic metal homeostasis and redox stability in *Brassica juncea*.

## 2. Materials and Methods

### 2.1. Plant Materials and Growth Conditions

*B. juncea* and *N. benthamiana* seeds (PI 211000) were used for transient expression. The seeds were washed with autoclaved distilled water and placed on wet blotting paper in Petri plates. The plates containing seeds were placed at 4 °C for 24 h for stratification. The next day, they were transferred to a climate-controlled room and incubated for 7 days at 22 °C under a 16 h day (100 µmol s^−1^ m^−2^) and 8 h night cycle at 70% relative humidity. *B. juncea* plants were then transferred to pots and allowed to grow for 15–20 d before infiltration [39]. *N. benthamiana plants* six- to 7-week-old post-germination, were used for agroinfiltration.

### 2.2. Cloning and Transformation of BjYSL6.1, BjYSL6.4 in Schizosaccharomyces pombe Vector pDES177N

*BjYSL 6.1* and *6.4* were cloned into the entry vector using BP reaction (Thermo Fisher Scientific, Waltham, MA, USA) and sequenced. The constructs were cloned into the *S. pombe* destination vector pDES177N as a fusion of GFP at the N terminus using the LR reaction. *S. pombe* cells were grown in YES medium. For each transformation 0.5–1 mL of cell was used. The cells were centrifuged, and the medium was removed from the cell pellet. Salmon sperm DNA (Sigma Aldrich, St. Louis, MO, USA) was denatured by heating at 90 °C for 5 min and placed on ice to keep it denatured. The cell pellet was resuspended in 0.5 mL PEGLET containing 5 μL salmon sperm DNA and vortexed to mix the solution. The construct (0.5 µg) was added to the destination vector and vortexed to mix. The cells were incubated at 28 °C overnight. The cells were centrifuged the next day, PEGLET was aspirated, 100 μL EMM medium was added, and the entire amount was spread on an EMM (−Uracil) selective plate. The plates were incubated for 3–5 days.

### 2.3. Confocal Microscopy of GFP: BjYSL6.1-3’UTR and GFP: BjYSL6.4-3’UTR Constructs in S. pombe

GFP: *BjYSL6.1-*3’UTR, GFP: *BjYSL6.4*-3’UTR, and empty vectors were transformed into *S. pombe.* Cells were inoculated into thiamine-containing minus uracil (−U) synthetic dropout medium and grown to saturation. Cells were washed and inoculated in fresh −U and minus Thiamine (−T) medium for induction of the nmt promoter present in the pDES177N vector at an O.D of 0.2 and then grown until saturation. Cells were stained with HOECSHT (5 µg/mL). The cells were observed under a confocal microscope (Olympus FluoView 1200, Olympus Corporation, Tokyo, Japan) using blue excitation (488 nm) and a green emission (509 nm) filter.

### 2.4. Interaction Between BjYSL6 Proteins with BjNRAMP4.1 and BjHCF164

The entry clone for *BjNRAMP4.1* was mobilized into the pMetYC_GW vector by LR reaction to create bait constructs. *BjNRAMP4.1* was transformed into *Saccharomyces cerevisiae* ThyAP4 cells. ThyAP4 cells containing *BjNRAMP 4.1* were co-transformed with either an empty prey vector (as an autoactivation control) or with *BjYSL6.1* and *BjYSL6.4* in pNXgate32-3HA [40], and transformants were selected on leucine and tryptophan (−L, −W) dropout media. Similarly, to study the interaction between BjYSL6.1-BjHCF164 and BjYSL6.4-BjHCF164, the entry clones for *BjYSL6.1* and *BjYSL6.4* were mobilized into the pMetYC_GW vector to create bait constructs. *BjYSL6.1* and *BjYSL6.4* were transformed into *Saccharomyces cerevisiae* ThyAP4 cells. ThyAP4 cells containing *BjYSL6.1* and *BjYSL6.4* were co-transformed with either an empty prey vector (as an autoactivation control) or with *BjHCF164* cloned in pNXgate32-3HA, and transformants were selected on −L−W media. The positive colonies were grown and spotted on histidine, tryptophan, methionine, and leucine dropout (−H, −W, −M, −L) interaction plates for four days. The agarose overlay assay was performed on yeast patches to select beta-galactosidase-positive colonies. The homodimeric interaction of the *Arabidopsis thaliana* potassium channel protein KAT1 (AT5G46240) was used as a positive control. ThyAP4 cells transformed with empty bait and prey vectors were used as negative controls.

### 2.5. β-Galactosidase Assay Using Agarose Overlay

Cells expressing both bait and prey fusions were spotted onto a solid interaction medium. Agarose (0.25 g) was dissolved in 50 mL Z-buffer (60 mM Na_2_HPO_4_.7 H_2_O, 40 mM NaH_2_PO_4_, 10 mM KCl, 1 mM MgSO_4_.7 H_2_O, pH 7.2) with moderate heating in a microwave oven. The agarose solution was cooled to 50 °C in a temperature-controlled water bath. 1 mL of 10% SDS, 0.3 mL β- mercaptoethanol and 1 mL of X-Gal stock solution was added to the Z-buffer agarose mix such that the final concentration of X-Gal was 1 mg/mL. The solution was carefully poured over the plate, and the plate was incubated at 37 °C. The plates were monitored for 15 min to 24 h, depending on the constructs.

### 2.6. Agrobacterium Transformation

Agrobacterium cells (1.5 mL) grown overnight were centrifuged at 14,000 rpm for 10 min at 4 °C. The supernatant was discarded, and the pellet was resuspended in 1 mL of ice-cold 20 mM CaCl_2_ solution. The cells were centrifuged again at 14,000 rpm for 5 min at 4 °C, followed by resuspension in 200 μL of ice-cold 20 mM CaCl_2_. The desired gene was cloned into suitable vectors and used for transformation. Constructs (0.5–1 μg) were added to the cell suspension and mixed by pipetting. The microcentrifuge tubes containing the cell suspension were frozen in liquid nitrogen for 5 min, thawed at 37 °C in a water bath for 5 min, and then cooled on ice. YEB medium (1 mL) was added to each tube and incubated at 28 °C for 2–5 h with gentle shaking. Finally, 50–200 μL of the cells were spread on YEB medium containing appropriate antibiotics and incubated at 28 °C for 2 days. To verify successful transformation, several colonies were randomly selected for plasmid isolation and subjected to PCR using gene-specific primers.

### 2.7. Localization of BjYSL6.1 and BjYSL6.4 in Brassica Determined by Confocal Microscopy

The *BjYSL6.1* and *BjYSL6.4* genes were mobilized into the pMDC45 vector [41,42]. GFP: *BjYSL6.1* and GFP: *BjYSL6.4* were transformed into *Agrobacterium tumefaciens* and selected on YEB plates containing kanamycin (50 μg/mL). *Agrobacterium tumefaciens* grown overnight at 28 °C in YEB medium was diluted to a final O.D_600_ of 0.2 with 10 mM MES-NaOH pH 6.2, 10 mM MgCl_2_, and acetosyringone (150 μM) and mixed with *AvrPto1* (final O.D: 0.1). The *AvrPto1* gene was cloned into the pXCSGStrep vector [43] and transformed into *A. tumefaciens* strain GV3101. *B. juncea* cotyledonary leaves were infiltrated as described in Das, Naiya, Marik, Mukherjee and Seal [39] and observed under Confocal laser scanning microscope (Olympus FluoView 1200, Olympus Corporation, Tokyo, Japan) was performed 48 h post-infection at an excitation wavelength of 473 nm and emission wavelength of 503–519 nm for GFP.

### 2.8. Bimolecular Fluorescence Complementation (BiFC) Assay to Study Interaction in Planta

For leaf expression, the plasmids containing the genes of interest were cloned into pSITE-nEYFP-C1 and pSITE-cEYFP-N1 vectors and transformed into *A. tumefaciens* for BiFC studies in plants (*Brassica juncea* and *Nicotiana benthamiana*). Transformed *A. tumefaciens* (GV3101) was selected on YEB agar plates containing 100 μg/mL of carbenicillin and 50 μg/mL of rifampicin. *A. tumefaciens* expressing *BjYSL6s*, *BjNRAMP4.1* and *BjHCF164* as fusions of the split YFP were grown overnight at 28 °C in YEB medium and resuspended in infiltration buffer (10 mM MES-NaOH pH 6.2 and 10 mM MgCl_2_), acetosyringone (150 μM, Himedia, Mumbai, India). The constructs were mixed with *Agrobacterium* cells expressing *AvrPto1* (for *B juncea* final O.D. 0.1) [39] or p19 (for *N. benthamiana* final O.D. 1). For transformation in *B. juncea/N. benthamiana*, each construct was diluted to a final O.D_600_ of 0.2 and 0.75, respectively. *B. juncea* leaves were infiltrated with mixed cultures of *BjYSL6.1/BjYSL6.4* and *BjNRAMP4.1* or *BjYSL6.1/BjYSL6.4* and *BjHCF164* along with *AvrPto1*. The leaves were observed under a confocal microscope 48 h post-infection (hpi). Mixed cultures of *BjNRAMP4.1* and *BjHCF164* were used to infiltrate *B. juncea* leaves and observed under a confocal microscope 72 hpi at an excitation wavelength of 473 nm and emission wavelength of 520–540 nm. *N. benthamiana* leaves were infiltrated with the mixed cultures expressing BjYSL6.1/BjYSL6.4 and BjNRAMP4.1 or BjYSL6.1/BjYSL6.4 and BjHCF164 along with p19. Leaves were observed under a confocal microscope 48 hpi. Similarly, mixed cultures expressing BjNRAMP4.1 and BjHCF164 were used to infiltrate *N. benthamiana* leaves and observed under a confocal microscope 72 hpi at an excitation wavelength of 473 nm and emission wavelength of 520–540 nm.

### 2.9. GUS Staining of Transiently Transformed N. benthamiana Leaves

*Nicotiana benthamiana* leaves were co-infiltrated with *BjYSL6.1* or *BjYSL6.4* constructs cloned into the pMDC140-GUS vector [41] together with the p19 silencing suppressor. The infiltrated leaves were incubated under standard growth conditions for 48–60 h post-infiltration. For histochemical analysis, the leaves were vacuum infiltrated with GUS staining buffer as described by Blume and Grierson [44]. After staining, chlorophyll was removed using 70% ethanol until the tissue became transparent. The samples were then examined under bright-field microscopy using a Nikon Eclipse Ts2R microscope (Nikon Corporation, Tokyo, Japan) to visualize GUS activity.

### 2.10. qRT-PCR PCR of BjYSL6 and BjNRAMP4.1 Gene

Total RNA was isolated from the shoots of *B. juncea* plants grown in Murashige and Skoog (MS) medium (control condition), MS medium plus 2 mM Fe and 2 mM Mn (+Fe, +Mn) or MS medium minus Fe or Mn (−Fe, −Mn). Expression analysis was performed using shoot tissues, as chloroplasts, the primary site of photosynthesis and metal-dependent redox processes, are predominantly present in aerial tissues, making them the most relevant system to study the functional coordination of *BjYSL6* and *BjNRAMP4.1*.

Total RNA was isolated from frozen *Brassica* shoot tissues using the HiPurATM Plant and Fungal RNA Miniprep Purification Spin Kit (Himedia, India). RNA was treated with DNase I (1Units/2 μg of RNA) to remove genomic DNA contamination. The integrity of the RNA was checked using formaldehyde agarose gel electrophoresis. To study the expression of metal transporter genes, 5-day old plants grown under +Fe, +Mn, −Fe, and −Mn conditions were compared with untreated controls. RNAs were treated with DNase to remove genomic DNA contamination. A Minus Reverse Transcriptase control was performed with real-time PCR primers (Table S1) to check for the removal of contaminating DNA. Primer sequences and PCR conditions are listed in Supplementary Table S1. Single bands of the PCR products for *BjNRAMP4.1*, *BjYSL6.1*, and *BjYSL6.4* specific real-time primers showed that the cDNAs were ideal for use in RT-PCR. Two micrograms of total RNA were used to synthesize cDNA with Random Hexamer Primer (Fermentas) using RevertAidTM Reverse Transcriptase (Fermentas 200 units/2 µg of RNA) according to the manufacturer’s protocol. The qRT-PCR reaction contained 5 μL DyNAmo ColorFlash SYBR Green I master mix (Thermo Scientific, Waltham, MA, USA), 0.5 μM of each primer, and 25 ng of cDNA. Real-time PCR was performed using a StepOne Plus real-time PCR machine (Applied Biosystems, Thermo Scientific, Waltham, MA, USA). The thermal step-up was 10 min at 95 °C, followed by 40 cycles of 10 s at 95 °C, 30 s at 65 °C. Data analysis was performed using the Expression Suite Software v1.0.4 (Applied Biosystems, Thermo Scientific, Waltham, MA, USA). Fold change in expression was calculated using the 2^−ΔΔCt^ method and plotted relative to the control using actin as a reference gene using GraphPad Prism 5.02. Experiments were performed in 3 biological replicates in triplicate. Statistical analysis was performed by Two-way ANOVA followed by Tukey’s multiple comparisons test. Statistical significance was set at *p* < 0.05. Different letters indicate significant differences between groups. All data are presented as mean ± SE.

## 3. Results

### 3.1. BjYSL6.1 and BjYSL6.4 Are Homologs of Arabidopsis Thaliana YSL6 Protein AtYSL6

*BjYSL6.1* and *BjYSL6.4* are two closely related isoforms or allelic variants cloned from the allopolyploid plant *B. juncea*, which are homologs of *AtYSL6* [45]. The two proteins are of equal size and share a high degree of sequence similarity among themselves, with most of the sequence dissimilarities lying in the last exon and 3’UTR (Figure S1). BjYSL6.1 and BjNRAMP4.1 were found to interact with [39] a chlorophyll-localized tetratricopeptide repeat-containing protein kinase (KM409600, [39]). As AtYSL6 is found to localize in chloroplast [16], we studied the localization of BjYSL6.1 and BjYSL6.4 in plants.

### 3.2. BjYSL6.1 and BjYSL6.4 Express in the Endomembranes of Schizosaccharomyces pombe and Shoot Membrane, and Chloroplast of B. juncea

**Figure 1:**
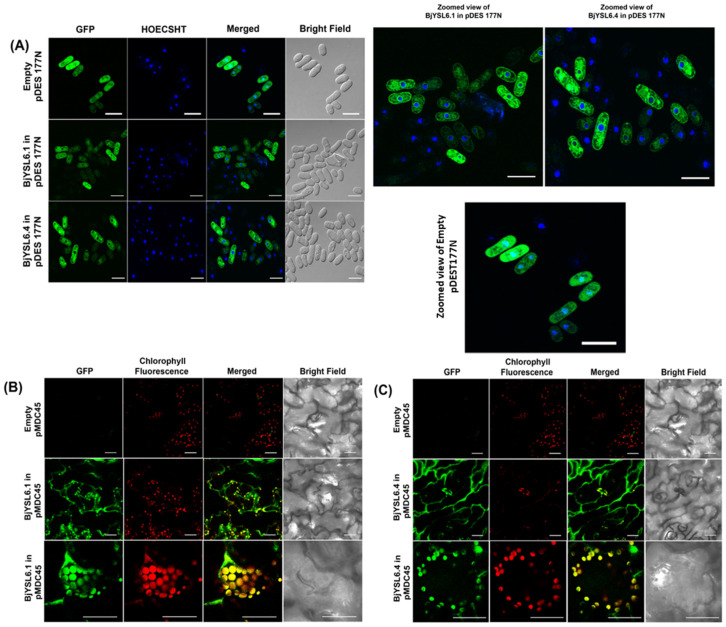
**BjYSL6.1 and BjYSL6.4 expressed in the endomembranes of *Schizosaccharomyces pombe* and leaf membrane and chloroplast of *Brassica juncea.*** (**A**) BjYSL6.1 and BjYSL6.4 are expressed in the endomembrane of *Schizosaccharomyces pombe*. To study the localization of *BjYSL6.1* and *BjYSL6.4*, the genes were fused to GFP and expressed in *S. pombe* cells. Confocal microscopy was performed. The upper panel shows an empty vector expressed in *S. pombe.* GFP (green) was expressed nonspecifically throughout the cells. *BjYSL6.1* (middle panel) and *BjYSL6.4* (lower panel) fused with GFP expressed at the endomembranes of *S. pombe* (Scale bar: 10 μm). The cells were counterstained with the nuclear stain DAPI (blue). A zoomed version of the upper, middle and lower panels is shown (right). (**B**) BjYSL6.1 and (**C**) BjYSL6.4 localize to the leaf membrane and chloroplast. GFP–*BjYSL6.1* and GFP–*BjYSL6.4* were expressed in *B. juncea* leaves by *A. tumefaciens-*mediated transient expression and imaged using confocal microscopy. (**B**) Upper panel: Expression of empty pMDC45. Middle panel: GFP–BjYSL6.1 expressed in the plasma membrane and chloroplast. Lower panel: GFP–BjYSL6.1 expressed in the chloroplast (Scale bar: 30 μm) (**C**) Upper panel: empty pMDC45 is expressed. Middle panel: GFP–BjYSL6.4 expresses in the plasma membrane. Lower panel: GFP–*BjYSL6.4* expresses in the chloroplast (Scale bar: 30 μm).

As previous studies have shown that members of the oligopeptide transporter (OPT) family can be mislocalized in *Saccharomyces cerevisiae* [46], we used *Schizosaccharomyces pombe* (strain GSY001) as a heterologous expression system to investigate the subcellular localization of BjYSL6.1 and BjYSL6.4. The coding sequences of both genes were fused with GFP, and their localization was visualized using confocal microscopy. Cells carrying the empty vector displayed diffuse fluorescence throughout the cytoplasm, indicating a nonspecific distribution of GFP. In contrast, fluorescence from BjYSL6.1-GFP and BjYSL6.4-GFP was distinctly confined to the plasma membrane and endomembrane system, including the nuclear envelope (Figure 1A; zoomed views). To validate these observations in planta, *BjYSL6.1*-GFP and *BjYSL6.4*-GFP were transiently expressed in *Brassica juncea* leaves using *Agrobacterium tumefaciens*–mediated infiltration, following a method optimized in our laboratory [39]. Both fusion proteins were predominantly localized to the plasma membrane and chloroplasts, as indicated by the overlap between the GFP and chlorophyll autofluorescence signals (Figure 1B,C).

### 3.3. BjYSL6.1 and BjYSL6.4 Exhibit Distinct Interaction Patterns with BjNRAMP4.1 and BjHCF164 in Yeast Two-Hybrid Assays

Previous work using the split-ubiquitin-based yeast two-hybrid (Y2H) system demonstrated that the Mn influx transporter BjNRAMP4.1 from *Brassica juncea* localizes to the chloroplast and interacts with the membrane-bound thioredoxin BjHCF164, a homolog of *Arabidopsis* HCF164 [29]. The Split-Ubiquitin Y2H system is specifically designed to detect interactions involving membrane proteins. In this system, the bait protein is fused to the C-terminal half of ubiquitin (Cub), whereas the prey protein, either cytosolic or membrane-bound, is fused to a mutated N-terminal half of ubiquitin (NubG). Upon bait-prey interaction, the two halves reconstitute functional ubiquitin, triggering the release of an artificial transcription factor (Protein A-LexA-VP16) that activates reporter genes [47].

**Figure 2:**
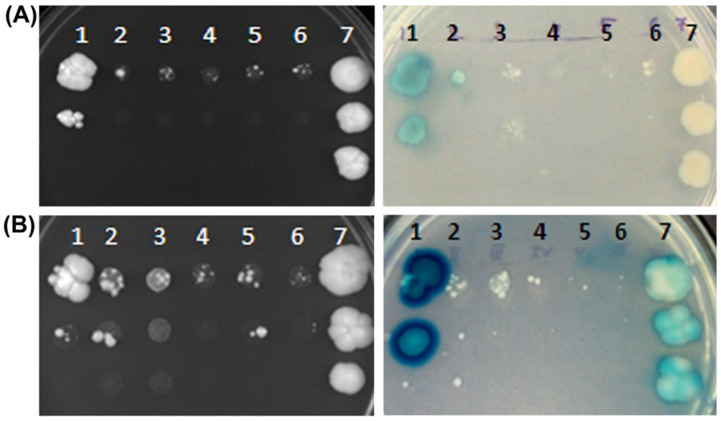
**Split Ubiquitin based Yeast Two-hybrid assay shows that both *BjYSL6.1* and *BjYSL6.4* interacts with *BjNRAMP4.1* and *BjHCF164*, but the level of interaction greatly varies depending on its partner protein** (**A**) *BjYSL6.1* or *6.4* and *BjNRAMP4.1* were cotransformed in ThyAP4 yeast cells and selected on interaction (−H−W−M−L) plate. 1. NubG*-BjYSL6.1*- + *BjNRAMP4.1-*Cub, 2. NubG-*BjYSL6.4* + *BjNRAMP4.1*-Cub, 3. Empty pNX32+ *BjNRAMP4.1-*Cub, 4. NubG*-BjYSL6.1* + empty pMetYC, 5. NubG*-BjYSL6.4* + empty pMetYC 6. Empty pNX32+ empty pMetYC, 7. *KAT1*-NubG + *KAT1*-Cub. Left panel: BjNRAMP4.1 and BjYSL6.1/6.4 interaction; Right panel: X-Gal overlay assay of BjNRAMP4.1 and BjYSL6.1/6.4 interaction. (**B**) *BjYSL6.1* or *6.4* and *BjHCF164* were co-transformed into ThyAP4 yeast cells and selected on an interaction (−H−W−M−L) plate. 1. *BjYSL6.1*-Cub + NubG*-BjHCF164*, 2. *BjYSL6.4*-Cub + NubG*-BjHCF164*, 3. Empty pMetYC + NubG*-BjHCF164*, 4. *BjYSL6.1*-Cub+ empty pNX32, 5. *BjYSL6.4*-Cub+ empty pNX32, 6. Empty pMetYC + empty pNX32. 7. *KAT1-*NubG + *KAT1*-Cub. Left panel: BjHCF164 and BjYSL6.1/6.4 interaction; Right panel: X-Gal overlay assay of BjHCF164 and BjYSL6.1/6.4 interaction.

To investigate whether BjYSL6.1 and BjYSL6.4 interact with BjNRAMP4.1 and BjHCF164, the corresponding genes were cloned into prey vectors and co-transformed with *BjYSL6.1* or *BjYSL6.4* bait constructs into *Saccharomyces cerevisiae* strain THYAP4. Transformed colonies were selected on quadruple dropout (−H, −L, −M, −W) plates and analyzed by spotting and X-gal overlay assays. A strong blue coloration indicated a positive interaction between BjYSL6.1 and BjNRAMP4.1, whereas BjYSL6.4 showed only a weak interaction (Figure 2A). Similarly, a clear interaction was observed between BjYSL6.1 and BjHCF164, but little or no interaction was detected between *BjYSL6.4* and *BjHCF164* (Figure 2B). Potassium channel KAT1 homodimerization was used as a positive control.

Although BjYSL6.1 and BjYSL6.4 share more than 90% amino acid sequence similarity, differing mainly in their final exon and 3′ UTR (Figure S1), their interaction patterns with partner proteins varied markedly. These results suggest that subtle sequence divergence between the two YSL6 isoforms strongly influences their binding specificity in yeast.

### 3.4. BjYSL6.1 and BjYSL6.4 Interact with BjNRAMP4.1 and BjHCF164 in Brassica juncea Leaf Albeit Differentially

To examine protein–protein interactions in planta, Bimolecular Fluorescence Complementation (BiFC) assays were performed. The genes were cloned into BiFC vectors pSITE-nEYFP-C1 and pSITE-cEYFP-N1, and the resulting constructs were co-infiltrated into *Brassica juncea* leaves, as described by Das et al. [39]. Confocal microscopy was performed 48 h post-infiltration to visualize YFP fluorescence as a sign of interaction. Chlorophyll autofluorescence was observed as a chloroplast marker.

**Figure 3:**
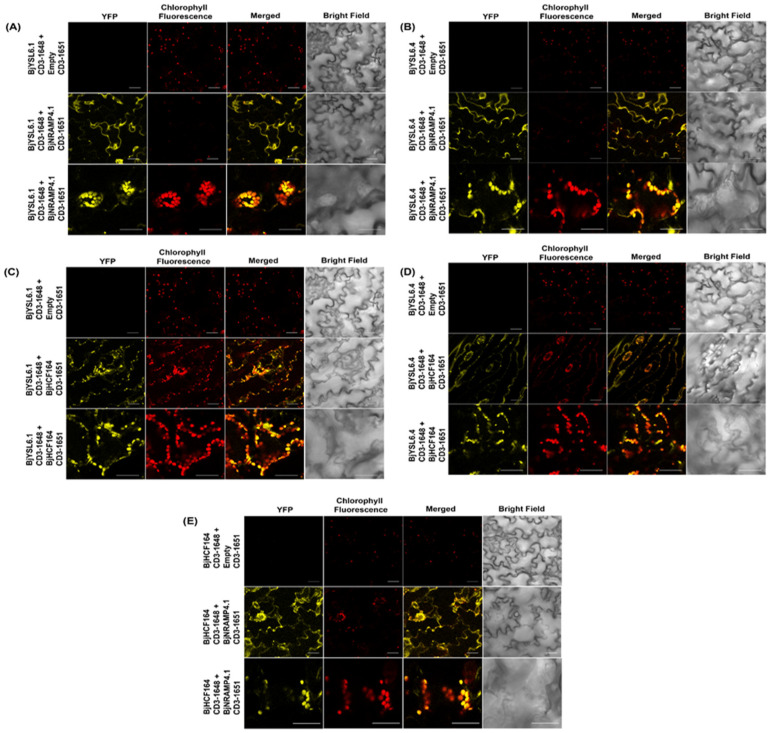
**BjYSL6.1 or BjYSL6.4 and BjNRAMP4.1 interact in chloroplast and cell membrane and BjYSL6.1 or BjYSL6.4 and BjHCF164 interact in the chloroplast of *Brassica juncea* leaves.** *BjYSL6.1*-nYFP, *BjYSL6.4*-nYFP, and *BjNRAMP4.1*-cYFP were expressed in *Brassica juncea* leaves for the BiFC assay and imaged using confocal microshlorophyll fluorescence was used to monitor co-localization in chloroplasts. (**A**,**B**) BjYSL6.1 or BjYSL6.4 and BjNRAMP4.1 interact in the chloroplast. (**A**) Upper panel: Leaves transformed with *BjYSL6.*1-nYFP and empty vector-cYFP; Middle panel: BjYSL6.1-nYFP and BjNRAMP4.1-cYFP interact at the plasma membrane (yellow fluorescence); Lower panel: BjYSL6.1-nYFP and BjNRAMP4.1-cYFP interact in chloroplasts (yellow fluorescence of YFP colocalized with red chlorophyll fluorescence) (Scale bar 30 µm). (**B**) Upper panel: Leaves transformed with *BjYSL6.4*-nYFP and empty vector-cYFP; Middle panel: BjYSL6.4-nYFP and BjNRAMP4.1-cYFP interact at the plasma membrane; Lower panel: BjYSL6.4-nYFP and BjNRAMP4.1-cYFP interact in chloroplasts (Scale bar 30 µm). (**C**,**D**) BjYSL6.1 or BjYSL6.4 and BjHCF164 interact in the chloroplast. (**C**) Upper panel: Leaves transformed with *BjYSL6.1*-nYFP and empty vector-cYFP. Middle panel: *BjYSL6.1*-nYFP and *BjHCF164*-cYFP interact in chloroplast. Lower panel: *BjYSL6.1*-nYFP and *BjHCF164*-cYFP also interacted in chloroplasts (zoomed view, scale bar = 30 µm). (**D**) Upper panel: Leaves transformed with *BjYSL6.4*-nYFP and empty vector-cYFP; Middle panel: BjYSL6.4-nYFP and BjHCF164-cYFP interact at the chloroplast. Lower panel: BjYSL6.4-nYFP and BjHCF164-cYFP also interact in chloroplasts (zoomed view; scale bar = 30 µm). (**E**) *BjNRAMP4.1* and *BjHCF164* interact in the chloroplast and cell membrane of leaves. *BjHCF164*-nYFP and *BjNRAMP4.1*-cYFP were expressed in *Brassica* leaves for the BiFC assay and imaged using confocal microscopy. Upper panel: Leaves transformed with *BjHCF164*-nYFP and empty vector-cYFP. Middle panel: BjHCF164-nYFP and BjNRAMP4.1-cYFP interact at the plasma membrane. Lower panel: BjHCF164-nYFP and BjNRAMP4.1-cYFP interact in chloroplasts (scale bar = 30 µm).

BjYSL6.1 and BjYSL6.4 both interacted with BjNRAMP4.1, showing YFP fluorescence at the plasma membrane and in chloroplasts (Figure 3A,B). The co-localization of YFP and chlorophyll fluorescence confirmed chloroplasts as one of the primary sites of interaction. The interactions between BjYSL6.1, BjYSL6.4, and BjHCF164 were also analyzed under identical conditions. Both YSL proteins interacted with BjHCF164, primarily in chloroplasts (Figure 3C,D). The punctate YFP signals observed at lower magnification (Figure 3C,D, middle panels) largely overlapped with the chlorophyll signal, indicating interaction foci within the chloroplast. No YFP fluorescence was detected in the negative controls, confirming the specificity of the assay. Consistent with our previous observations [29], BjNRAMP4.1 and BjHCF164 also interacted at both the plasma membrane and within chloroplasts (Figure 3E).

### 3.5. BjYSL6.1 and BjYSL6.4 Interact with BjNRAMP4.1 and BjHCF164 in Nicotiana benthamiana Leaves

To confirm the protein–protein interactions observed in *Brassica juncea*, we revalidated them in the model plant *Nicotiana benthamiana*. This species is widely used for transient expression assays to analyze subcellular protein localization and protein–protein interactions without requiring stable transformation [48]. For efficient expression in leaves, all constructs were co-infiltrated with the p19 suppressor of post-transcriptional gene silencing (PTGS).

**Figure 4:**
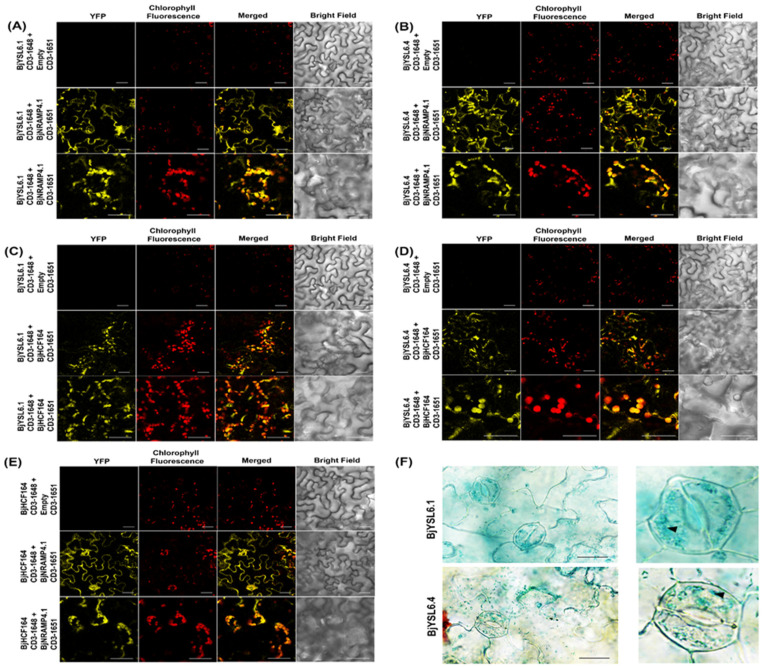
**BjYSL6.1 and BjYSL6.4 interact with BjNRAMP4.1 in the chloroplast and plasma membrane, and BjYSL6.1 and BjYSL6.4 interact with BjHCF164 in the chloroplast of *Nicotiana benthamiana* leaves.** *BjYSL6.1*-nYFP, *BjYSL6.4*-nYFP, and *BjNRAMP4.1*-cYFP were expressed in *Nicotiana benthamiana* leaves for BiFC assay and imaged by confocal microscopy. Chlorophyll fluorescence was used to monitor co-localization in chloroplasts. (**A**,**B**) BjYSL6.1 or BjYSL6.4 and BjNRAMP4.1 interact in the chloroplast. (**A**) Upper panel: Leaves transformed with *BjYSL6.1*-nYFP and empty vector-cYFP; Middle panel: BjYSL6.1-nYFP and BjNRAMP4.1-cYFP interact at the plasma membrane (yellow fluorescence); Lower panel: BjYSL6.1-nYFP and BjNRAMP4.1-cYFP interact in chloroplasts (Scale bar 30 µm). (**B**) Upper panel: Leaves transformed with *BjYSL6.4*-nYFP and empty vector-cYFP; Middle panel: BjYSL6.4-nYFP and BjNRAMP4.1-cYFP interact at the plasma membrane; Lower panel: *BjYSL6.4*-nYFP and *BjNRAMP4.1*-cYFP interact in chloroplasts (yellow fluorescence of YFP colocalized with red chlorophyll fluorescence) (Scale bar 30 µm). (**C**,**D**) BjYSL6.1 or BjYSL6.4 and BjHCF164 interact in the chloroplast. (**C**) Upper panel: Leaves transformed with *BjYSL6.*1-nYFP and empty vector-cYFP; middle panel: BjYSL6.1-nYFP and BjHCF164-cYFP interact in the chloroplast; lower panel: *BjYSL6.1*-nYFP and *BjHCF164*-cYFP also interact in the chloroplasts (zoomed view, scale bar = 30 µm). (**D**) Upper panel: Leaves transformed with *BjYSL6.4*-nYFP and empty vector-cYFP; Middle panel: BjYSL6.4-nYFP and BjHCF164-cYFP interact at the chloroplast. Lower panel: BjYSL6.4-nYFP and BjHCF164-cYFP also interact in chloroplasts (zoomed view; scale bar = 30 µm). (**E**) BjNRAMP4.1 and BjHCF164 interact in the chloroplast and plasma membrane of leaves. *BjHCF164*-nYFP and *BjNRAMP4.1*-cYFP were expressed in *Nicotiana benthamiana* leaves for the BiFC assay and imaged using confocal microscopy. Upper panel: Leaves transformed with *BjHCF164*-nYFP empty vector-cYFP. Middle panel: BjHCF164-nYFP and BjNRAMP4.1-cYFP interactions at the plasma membrane; Lower panel: BjHCF164-nYFP and BjNRAMP4.1-cYFP interactions in chloroplast (yellow fluorescence of YFP colocalized with red chlorophyll fluorescence). (Scale bar 30 µm). (**F**) BjYSL6.1 and BjYSL6.4 proteins fused with β-glucuronidase (GUS) were expressed in chloroplasts. BjYSL6.1 and BjYSL6.4 proteins were fused to the GUS protein and expressed in *N. benthamiana* leaves. The leaves were infiltrated with GUS staining buffer, destained, and observed under a microscope (scale bar = 10 µm). Arrowheads indicate blue stains in chloroplasts in the zoomed-in images (right side).

Confocal microscopy was performed 48 h post-infiltration to visualize yellow fluorescent protein (YFP) fluorescence and chlorophyll autofluorescence. Both BjYSL6.1 and BjYSL6.4 interacted with BjNRAMP4.1, with YFP fluorescence detected at the plasma membrane and within the chloroplasts (Figure 4A,B), consistent with their interaction patterns in *B. juncea*. Overlapping YFP and chlorophyll fluorescence signals confirmed the chloroplast localization of the interaction sites.

Similarly, interactions between BjHCF164 and either BjYSL6.1 or BjYSL6.4 produced strong YFP fluorescence, which was primarily confined to the chloroplasts (Figure 4C,D). The punctate YFP signals observed at lower magnification largely colocalized with chlorophyll fluorescence, suggesting the presence of interaction foci within the chloroplasts. No fluorescence was detected in the negative controls, confirming the assay specificity.

Consistent with previous reports [29], BjNRAMP4.1 and BjHCF164 interacted in both the plasma membrane and chloroplast compartments (Figure 4E). These findings indicate that BjYSL6 isoforms are associated with distinct chloroplast and membrane partners in a compartment-specific manner.

To further validate chloroplast localization, *BjYSL6.1* and *BjYSL6.4* were fused at their C-termini with the GUS reporter and transiently expressed in *N. benthamiana* leaves. GUS staining revealed blue coloration within the chloroplasts of guard cells, confirming the chloroplast localization of the BjYSL6.1-GUS and BjYSL6.4-GUS fusion proteins (Figure 4F).

### 3.6. BjYSL6.1, BjYSL6.4, and BjNRAMP4.1 Exhibit Opposite Transcriptional Expression Under Metal Excess and Deficiency Conditions

**Figure 5:**
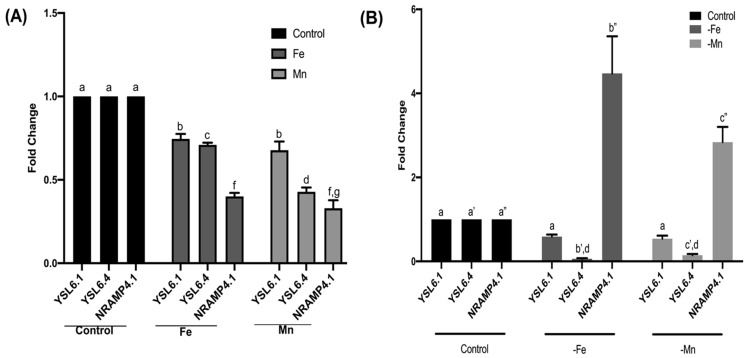
Expression of *BjNRAMP4.1*, *BjYSL6.1* and *BjYSL6.4* in presence of excess and absence of essential metals. Expression of *YSL* and *NRAMP* was studied using qRT-PCR using RNA from control plants and plants treated with (+Fe, +Mn) or without iron and manganese (−Fe, −Mn). (**A**) Expression of *BjYSL6.1*, *BjYSL6.4*, and *BjNRAMP4.1* in Fe (2 mM) and Mn (2 mM)-treated plants. (**B**) Expression of *YSL* and *NRAMP* in −Fe and −Mn plants. Gene expression levels were calculated as fold change relative to the control, which was normalized to 1. Statistical analysis was performed using two-way ANOVA followed by Tukey’s multiple comparisons test, with significance set at *p* < 0.05. Different lowercase letters indicate statistically significant differences among groups. Data are presented as mean ± SE (*n* = 3 biological replicates). The control condition represents the normalized reference baseline and therefore does not display variability or error bars.

Because *BjYSL6* transporters and *BjNRAMP4.1* are involved in Fe and Mn homeostasis, we examined how their expression levels respond to varying metal conditions. Total RNA was isolated from the shoots of *Brassica juncea* plants grown under control conditions (MS medium) or supplemented with 2 mM Fe or 2 mM Mn (+Fe or +Mn). Under excess metal conditions, all three transporters, *BjYSL6.1*, *BjYSL6.4*, and *BjNRAMP4.1*, were downregulated compared to the control. However, the reduction in expression was less pronounced for *BjYSL6.1* and *BjYSL6.4* than for *BjNRAMP4.1*, suggesting a differential transcriptional sensitivity of these genes to Fe and Mn excess conditions(Figure 5A).

To further assess their metal-dependent regulation, plants were grown under Fe- or Mn-deficient conditions (−Fe, −Mn). Under these conditions, the expression of *BjNRAMP4.1* was strongly upregulated, whereas that of *BjYSL6.1* and *BjYSL6.4* was downregulated relative to the control (Figure 5B). This inverse regulation pattern indicates that *BjNRAMP4.1* functions predominantly as an influx transporter activated during Fe or Mn deficiency, whereas *BjYSL6.1* and *BjYSL6.4* likely function as efflux or redistribution transporters that are repressed when external metal availability is low.

## 4. Discussion

Chloroplasts possess a complex internal architecture consisting of a double membrane envelope, stroma where carbon fixation occurs, and thylakoid membrane system that drives photosynthetic electron transport. Proteomic studies have identified more than 90 potential transporters within the chloroplast envelope that supply metal ions and metabolites to sustain chloroplast function [49], although the roles of most remain poorly understood. Oligopeptide and yellow stripe-like (YSL) transporters are associated with distinct functions in long-distance metal circulation, nitrogen assembly, metal sequestration, and glutathione transport, and have been widely implicated in Fe, Zn, and Mn mobilization in plant tissues [10]. The localization of *Arabidopsis* YSL6 to chloroplasts has been debated since Divol et al. [16] suggested its presence in the chloroplast envelope, although this view remains contested [49]. Mutation in a *YSL* gene in cucumber results in a decline in chloroplast-related genes and a yellow cotyledon phenotype [50].

Here, we demonstrate that two *Brassica juncea* homologs, *BjYSL6.1* and *BjYSL6.4*, share functional similarities with *Arabidopsis* YSL6 and localize to the chloroplast. In addition, both isoforms were observed at the plasma membrane in transient expression assays. While both *BjYSL6* isoforms localize to the plasma membrane and chloroplasts, the present study does not quantify their relative abundance in these compartments. Future studies using quantitative imaging or biochemical fractionation will be required to determine their distribution ratios. Previous work by Divol et al. [16] reported that AtNRAMP4 protein levels decreased in the *ysl4ysl6* double mutant, implying that AtYSL4 and AtYSL6 regulate AtNRAMP3/4 abundance. Our results are consistent with this relationship, showing that BjYSL6.1 and BjYSL6.4 interact with the chloroplastic Mn transporter BjNRAMP4.1 and membrane-bound thioredoxin BjHCF164. While yeast two-hybrid assays indicated weak interaction in certain combinations, BiFC assays in planta demonstrated detectable interactions. The discrepancy between yeast two-hybrid and BiFC results likely reflect differences in membrane composition, cellular context, and protein folding, highlighting the importance of validating protein interactions in planta. In *Schizosaccharomyces pombe*, the fluorescence patterns of *BjYSL6.1* and *BjYSL6.4* suggest localization throughout the endomembrane system, indicating potential trafficking through the secretory pathway.

Chloroplast proteins are generally nuclear-encoded and post-translationally imported via TOC/TIC translocon complexes [51]. However, alternative vesicular routes have been proposed, allowing specific nuclear-encoded proteins to reach chloroplasts independently of the classical import pathways [52]. Our findings that BjYSL6.1 and BjYSL6.4 localize to chloroplasts despite lacking canonical transit peptides suggest that they may use a secretory or vesicle-mediated trafficking route for chloroplast import. Indeed, several plasma membrane and vacuolar proteins, such as Plasma Membrane Intrinsic Proteins (PIPs) and Tonoplast Intrinsic Proteins (TIPs), have been identified in chloroplast proteomes [53,54], indicating that limited inter-organelle trafficking between endomembranes and chloroplasts may not be uncommon.

We previously showed that BjNRAMP4.1 localizes to the chloroplast and interacts with BjHCF164, a thylakoid membrane–bound thioredoxin-like protein [29]. The *Arabidopsis* ortholog AtHCF164 is essential for cytochrome b6f complex assembly and mediates redox regulation of the LHCII kinase STN7 via the reduction of luminal cysteine residues. HCF164 integrates short-term state transitions with long-term photosystem stoichiometry adjustments in response to environmental cues. Consistent with these functions, we observed that BjHCF164, BjNRAMP4.1, and the two BjYSL6 isoforms localized within chloroplasts and physically interacted. The overlap of GFP and chlorophyll fluorescence signals indicates that these interactions likely occur on the chloroplast envelope and thylakoid membranes, particularly within the stromal thylakoids [55,56].

Iron exporters such as Ferroportins (FPN3/IREG3) play complementary roles in maintaining organellar iron balance in plants. FPN3 functions as a dual-targeted exporter of Fe^2+^ from mitochondria and chloroplasts [57], underscoring that plants employ multiple efflux systems to prevent iron overaccumulation in organelles. The maintenance of organellar homeostasis, such as in chloroplasts, may necessitate the coordinated activity of both importers and exporters. In human functions of DMT1 a NRAMP iron importer protein is regulated by human ferroprotein counterpart [24].

Dual localization of metal transporters is not uncommon, and recent genome-wide analyses of YSL genes in *Nicotiana tabacum* further support their differential expression across tissues and subcellular compartments [13]. Dual expression systems in *B. juncea* and *Nicotiana benthamiana* further support the physiological relevance of these interactions. *N. benthamiana* is well established for high-level transient expression [58], whereas expression in *B. juncea* is lower, reducing the potential artifacts caused by ectopic overexpression. Using the native Brassica system ensured that all four proteins, *BjNRAMP4.1*, *BjYSL6.1*, *BjYSL6.4*, and *BjHCF164*, interacted within their physiological context.

Similar to BjYSL6 transporters, other iron exporters contribute to maintaining organellar iron balance. Notably, the *Arabidopsis* Ferroportin 3 (FPN3/IREG3) was recently identified as a dual-targeted iron exporter localized to both mitochondria and chloroplasts [57]. Loss of FPN3 causes iron accumulation in these organelles and triggers increased expression of YSL4/6 and NRAMP4, suggesting a compensatory relationship among iron efflux systems. These findings imply that YSL4/6 and FPN3 share overlapping roles in chloroplast iron export, but differ in substrate specificity. FPN3 exports ferrous ions (Fe^2+^), whereas YSL4/6 mediate efflux of Fe-nicotianamine complexes. The partial redundancy between these transporters highlights the existence of a coordinated iron efflux network that safeguards chloroplasts and mitochondria from metal-induced oxidative stress.

Recent proteomic analyses of chloroplast envelope fractions by Bouchnak et al. [55] identified AtHCF164, AtYSL6, and AtNRAMP4, among other low-abundance transporters. Despite stringent filtering that excluded AtYSL6 and AtNRAMP4 from their final dataset, the detection of all three proteins in their primary screen strongly supports the association of these proteins with chloroplasts. Overtly strict bioinformatic cutoffs may exclude transiently or loosely membrane-associated proteins, as evidenced by the absence of the known thylakoid transporter PAM71 in the same dataset. Interestingly, the cyanobacterial homolog SynPAM71 localizes to both the plasma membrane and thylakoids, mediating Mn export under toxic conditions [59], suggesting that the dual localization of metal transporters may be functionally conserved.

Our results support a model in which *BjNRAMP4.1*, *BjYSL6.1*, *BjYSL6.4*, and *BjHCF164* form a dynamic interaction network bridging the membrane, chloroplast envelope, and thylakoid membranes. Their spatial distribution and expression patterns suggest complementary roles for *BjNRAMP4.1* as an influx transporter, *BjYSL6s* as efflux or redistribution transporters, and *BjHCF164* as a photosynthetic redox regulator. The interplay among these components likely facilitates the fine-tuning of metal homeostasis, redox balance, and photosynthetic efficiency in *B. juncea*.

## 5. Conclusions

The chloroplast serves as the primary organelle for redox sensing, and both photosynthetic efficiency and chloroplastic redox balance are closely regulated by the availability of Fe and Mn. In this study, we demonstrated that the two yellow stripe-like transporters, BjYSL6.1 and BjYSL6.4, exhibit dual localization similar to the Mn influx transporter BjNRAMP4.1 and the membrane-bound thioredoxin BjHCF164, being present at both the plasma membrane and within chloroplasts. Protein–protein interaction studies revealed that BjNRAMP4.1, BjYSL6.1, and BjYSL6.4 interact at both the plasma membrane and chloroplasts, whereas BjHCF164 interacts primarily with BjYSL6.1 and BjYSL6.4 in chloroplasts. These results suggest that these proteins may dynamically associate and potentially shuttle between cellular membranes and chloroplasts in response to metabolic and redox cues. Such interactions likely form an integrated network linking metal homeostasis with photosynthetic regulation, thereby maintaining the optimal balance of essential transition metals within the photosynthetic machinery of *Brassica juncea*.

## 6. Limitations and Perspectives

In this study, we established the localization and interactions of BjYSL6.1, BjYSL6.4, BjNRAMP4.1, and BjHCF164 using complementary heterologous and in planta expression systems. Although the data provide strong evidence for their physical association and coordinated transcriptional responses to Fe and Mn status, the precise mechanistic sequence linking these interactions to metal flux and photosynthetic performance remains unclear. Future studies involving transgenic overexpression or knockdown lines will be important to establish the physiological roles of *BjYSL6* isoforms and their interaction network in planta. Also, quantitative approaches such as FRET or fluorescence intensity-based analysis will be valuable to determine the strength of these interactions.

The observed dual localization and co-regulation patterns suggest a functional interplay between metal transport and redox regulation; however, further biochemical or physiological studies are required to fully resolve their integrated roles. The present study provides a framework for understanding chloroplast-associated metal transport networks in *Brassica juncea*.

## Data Availability

*BJYSL6.1* (EU761239.2), *BJYSL6.4* (FJ529187.2), *BjNRAMP4.1* (EU839503.2) sequences are available in GenBank.

## Supplementary Materials

The following supporting information can be downloaded at: https://www.mdpi.com/article/10.3390/membranes16050167/s1. Figure S1: Full-length BjYSL6.1 and BjYSL6.4 were aligned using AlignX program of Vector NTI AdvanceTM 11, Boxed region in the alignment indicates the position of stop codons. Table S1: List of primers used in the study.
