# Supplementary figures and images for: A Metal Importer and Exporter Interact Differently in the Chloroplast and Cell Membrane
Source: Membranes (Basel). 2026 May 2;16(5):167. doi: 10.3390/membranes16050167 (PMC13208197; doi:10.3390/membranes16050167)

## Slide 1
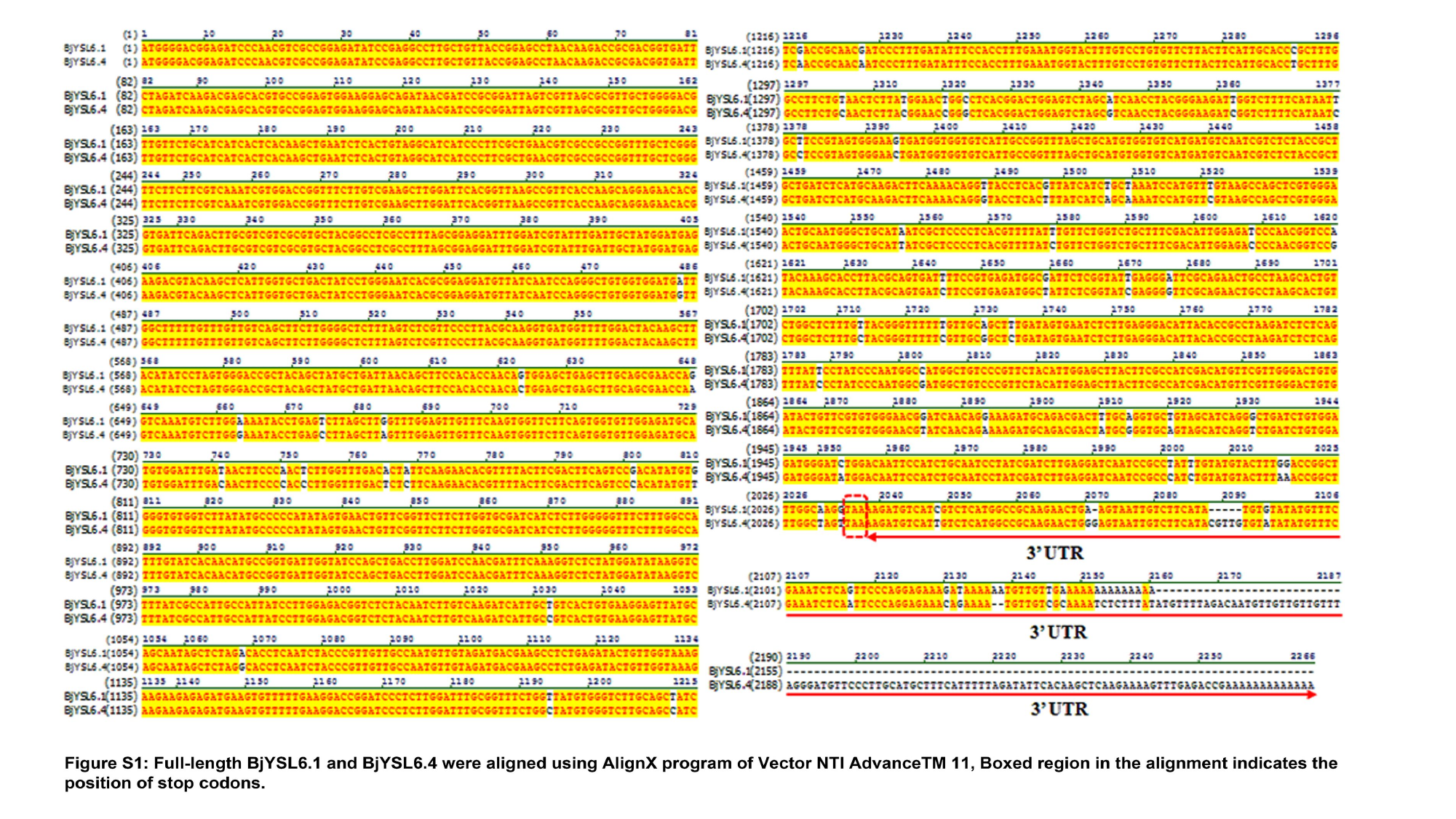

Supplement: Supplementary file 1 [file membranes-16-00167-s001.zip › Supp data.pptx]
